# The impact of the Qinghai-Tibet highway on plant community and diversity

**DOI:** 10.3389/fpls.2024.1392924

**Published:** 2024-06-28

**Authors:** ZhaoXian Tan, XuePing Chen, Yun Wang, Suqin Wang, Rong Wang, BaoHui Yao, YanGang Yang, YaPing Kong, JiaPeng Qu

**Affiliations:** ^1^ School of Life Science, Qinghai Normal University, Xining, China; ^2^ Sanjiangyuan Grassland Ecosystem National Observation and Research Station, Key Laboratory of Adaptation and Evolution of Plateau Biota, Northwest Institute of Plateau Biology, Chinese Academy of Sciences, Xining, China; ^3^ Academy of Plateau Science and Sustainability, People’s Government of Qinghai Province & Beijing Normal University, Beijing, China; ^4^ Research Center for Environment Protection and Water and Soil Conservation, China Academy of Transportation Sciences, Beijing, China; ^5^ Qinghai Province Key Laboratory of Animal Ecological Genomics, Northwest Institute of Plateau Biology, Chinese Academy of Sciences, Xining, China

**Keywords:** Qinghai-Tibet Plateau, road distance, species diversity, plant functional diversity, road ecology

## Abstract

Roads are an increasingly prevalent form of human activity that drives the decrease in plant community functions and threatens global biodiversity. However, few studies have focused on the changes in the function and diversity of alpine meadows caused by road infrastructure in the Tibetan Plateau. In this study, the changes in species diversity, functional diversity, and community stability were examined at different distances from the Qinghai-Tibet highway. The results showed that the road intensified the degradation of vegetation, which significantly altered species diversity and community structure. This effect gradually decreased from near to far from the highway. Plant community cover and species diversity were highest at intermediate distances (50–100 m) from the roadway; species diversity and stability were lowest in the grassland most disturbed by the road (0 m), and species diversity and functional diversity tended to stabilize farther away from the road (250 m). Our findings indicate that changes in species diversity are synchronized with changes in functional diversity, which largely determines the outcome of degraded grassland community diversity and stability. Our results provide a reference point for restoring degraded alpine areas and mitigating the ecological impacts of roads.

## 1 Introduction

Road networks are a ubiquitous phenomenon that creates a driving force from outside the ecosystem causing mortality or serious damage to organisms and changing the availability of resources (Mackey and Currie, 2000). Roads are the basic infrastructure that promotes social and economic progress, and the growth of road transportation is closely related to economic expansion (Meijer et al., 2018). Transportation infrastructure, such as roads, have become a common feature of the contemporary landscape (Skorobogatova and Kuzmina-Merlino, 2017), seemingly covering a small proportion of the land surface but impacting the ecological environment in various ways. Roads have huge ecological impacts, including modifying habitat, disrupting and eroding ecological flows, and increasing pollution (Asher et al., 2020). For example, the 6.3 million kilometers of roads in the United States only cover about 1% of the land, but they impact approximately 20% of the landscape (Forman and Deblinger, 2000). The Netherlands is also affected by the same percentage of roads (Reijnen and Foppen, 2006). Many other developed countries (e.g., the United Kingdom, Germany, and Japan) have road densities that are 2.5 to 4 times higher than those in the United States (Jaeger et al., 2007), with even worse environmental impacts. At least 25 million kilometers of new roads will be built worldwide by 2050; the total length of roads will increase by 60% compared to 2010, and transportation infrastructure will play an important role in shaping the environment (Meijer et al., 2018). The construction of roads represents a pervasive form of natural landscape transformation (Patarasuk and Binford, 2011). Economic development will require the construction of many new roads in the coming decades (Xie et al., 2021), which will extend into once inaccessible mountainous areas; consequently, these areas will face a balance between conservation of biodiversity and economic growth (Miller et al., 2021). Studies of various terrestrial and aquatic ecosystems have shown that many of the most prevalent threats to biodiversity (e.g., habitat destruction, fragmentation, edge effects, invasive alien species, pollution, overhunting, and genetics) are directly or indirectly related to roads (Lapaix et al., 2012), and attention has been paid to study the ecological characteristics of edges associated with roads (Jackson and Fahrig, 2011).

As typically linear corridors, roads serve as conduits to facilitate the movement of species between detached patches of remnant habitat, thereby promoting gene flow and increasing species richness (Lugo and Gucinski, 2000). Roads drive change in the composition and structure of mountain communities through habitat loss, fragmentation, and pollution from vehicles burning fossil fuels (Kitagawa et al., 2018). These changes affect biodiversity and influence ecosystem structure and function (De Pauw et al., 2021). Roads can change vegetation, including the loss of height or biomass, reduced cover, and changes in species composition (Pickering and Hill, 2007). Constructing and maintaining roads removes vegetation, changes land use (Saunders et al., 2002), alters soil structure and leads to an increase in soil compactness (Neher et al., 2013), indirectly affecting soil conditions. This, in turn, affects plant growth and species diversity, and promotes the establishment of invasive plants (Johnston and Johnston, 2004), resulting in the conversion of natural habitats to highly invasive roadside environments (Gelbard and Belnap, 2003). Formerly remote areas are becoming more accessible due to new roads, which not only cause a direct disturbance but also act as corridors for the movement of plant species (Rew et al., 2018). The role of roads as dispersal corridors is often amplified by vehicle traffic (Müllerová et al., 2011). The distance from the road and operating hours may also affect the abundance and distribution of native species. Roadside habitats provide ideal growing spaces for non-native plants, which often benefit from the more favorable microclimatic, the hydrological conditions, and the moderate disturbance (Averett et al., 2016). The success of native plant species fixation may be limited by the road and environmental conditions. For example, vegetation cover is minimal at roadsides but more abundant in interior areas (Cameron and Bayne, 2009).

The Tibetan Plateau is the world’s “third pole,” and has the largest area of perennial permafrost in the world (Pan et al., 2012). It is a natural barrier to the Asian water cycle and ecological security, and the alpine permafrost grasslands are experiencing climate change and anthropogenic disturbances (Lu et al., 2017). Climate extremes have made alpine grassland ecosystems more sensitive and fragile and particularly vulnerable to human activities (Hua and He, 2011), making restoration of grassland vegetation more challenging (Roy-Léveillée et al., 2014). Large-scale road development and excavation have artificially exacerbated erosion in recent years, leading to the degradation of perennial permafrost, to the detriment of the ecological security in the area. The Tibetan Highway crosses the Tibetan Plateau, a unique and fragile ecosystem. The Qinghai-Tibet Highway is an ideal place to study the effects of road construction and maintenance on species diversity (Zhao et al., 2006). Vegetation, which is the basis of this ecosystem, grows very slowly under localized environmental conditions. In addition, the interaction between anthropogenic disturbances and climate change makes it difficult to restore work in this region (Klein et al., 2004).

Roads are a major cause of the loss of biodiversity in mountainous areas worldwide (Li et al., 2022). Research into the effects of roads on species diversity in mountainous regions has gained momentum during the past two decades. A standardized protocol for monitoring changes in the altitudinal distribution, abundance, and composition of plant biodiversity in mountainous regions due to the interaction between the climate and human pressures has been proposed by previous authors. The protocol was developed by the Mountain Invasions Research Network (MIREN, www.mountaininvasions.org) (Rashid et al., 2021). Studies on the ecological effects of the Qinghai-Tibet Highway have focused on the observation that highway-related activities have exacerbated the degradation of vegetation along the route and have significantly changed the soil physicochemical properties (Liu et al., 2021). Changes in functional diversity and stability of the community structure have not yet been explored. However, the effects of changes in species composition and functional diversity in communities on ecosystem functioning may be equal to or stronger than the effects of changes in species richness (Shi et al., 2016). Plant functional trait diversity is sensitive to perturbations and environmental stress and is decisive for changes in ecosystem functioning. Therefore, assessing road-driven changes in species abundance and changes in functional diversity is critical to understanding the mechanism by which these compositional changes affect community and ecosystem functioning in response to road construction (Li et al., 2022). In addition, analyzing the response of β-diversity at different distances from the road and different elevations and describing the relationship between different diversities is important for understanding the mechanisms of community species coexistence on a landscape scale and conserving species, habitats, and natural ecological processes. Thus, we hypothesized that 1) plant communities at different distances from the road would be differently affected by the road, with plant richness being lower at the road edge, varying across the altitudinal gradient and influenced by climatic factors at the survey site; 2) lower plant functional richness would be detected at the edge of the road compared to different distances from the road; 3) plant community composition will differ by distance from the road, which may affect the plant community network structure. Our study will provide theoretical support for solutions to mitigate road-impacted grassland restoration on the Tibetan Plateau and to achieve mutual conservation benefits for humans and animals.

## 2 Materials and methods

### 2.1 Study sites

**Figure 1:**
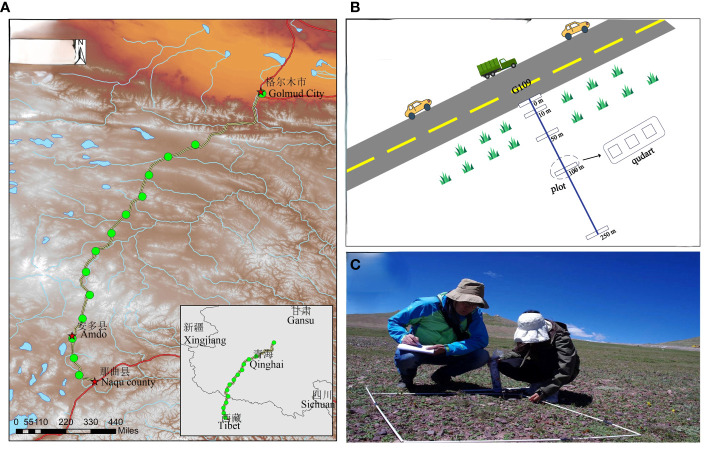
Schematic diagram of the distribution and survey of the 14 sample lines on National Highway 109. **(A)** Equal kilometer distribution of 14 sample sites along the road; **(B)** each sample site consists of five 2 m × 50 m plots parallel to the roadside. Plot 1- parallel to the roadside (starting at the first occurrence of roadside vegetation). Plots 2 – 5 represent varying distances from the road; **(C)** exemplary photograph of monitoring a mountain roadside on Qinghai Tibet highway, depicting a survey of plot 1.

The effect of the road on plant community diversity at the perennial permafrost zone side of State Route 109 on the Tibetan Plateau was investigated. After crossing the Tanggula Mountain area in the hinterland of the Tibetan Plateau on the 109 National Highway, the terrain is high in the west and low in the north, tilting from west to east (35°18′-36°23′E, 91°47′-94°55′N, **Figure 1**). The elevation ranges from 4,300 m to 5,135 m, with an average elevation of 4,500 m and many high mountainous peaks (Zhang et al., 2002). To explore the mechanism by which roads affect diversity across altitudinal segments, we categorized the elevations at our sampling sites into high, medium, and low groups for every 600 m. The typical vegetation of the region is highland subtropical semi-arid climate, and the vegetation is mainly alpine grassland dominated by *Stipa purpurea* and the alpine meadow is dominated by *Kobresia* spp. and *Carex* spp. The grassland communities on the side of the Qinghai-Tibet Highway were affected to varying degrees, with the slopes most affected by the highway and differing markedly from the vegetation composition of the interior grassland. Daytime traffic on the Qinghai-Tibet Highway is around 50 vehicles (Yin et al., 2006).

### 2.2 Field sampling design and survey

Field sampling was conducted during peak biomass and flowering in July–August 2022 to minimize the risk of missing species with early or late phenology. Typical sample strips were systematically set up in grassland communities on one side of the permafrost section of the Qinghai-Tibet Highway. The selected road began at the bottom of the mountainous area through which National Highway 109 passes and reached the highest elevation of the permafrost section of the Tibetan Plateau, with sample lines spaced at 50-km intervals, and the direction of the sample strips was perpendicular to the direction of the highway. Before entering the site to identify the samples, a global positioning system was used to locate the samples as accurately as possible, collect some basic environmental variables (e.g., soil characteristics, and topographic variables), and take photographs of each sample line. Highway mileage ranged from K2886 to K3537, with a total length of 650 km and sample line intervals of 50 km, with 14 sample lines in total. Five 2 m × 50 m sampling plots were set up parallel to the road on the sample line at different distances from the highway: 0 m from the highway (slopes), 10, 50, 100, and 250 m (the furthest distance that could be extended). Three 1 m × 1 m sample squares were laid out at each site (**Figure 1**). In each of the three sample quadrats at all sites, we investigated the abundance, coverage, and height of all native and non-native plants. Plant coverage was represented by the ratio of the shaded area of a species to the total area of the quadrat. Functional plant traits were recorded, including leaf shape, leaf texture, seed dispersal mode, life history, light habit, and growth environment. Unrecognized plants were identified to the species level using the most up-to-date local flora.

### 2.3 Data analysis

#### 2.3.1 Biodiversity measures

α-Diversity and β-diversity were selected to characterize the spatial dynamics of biodiversity in the highway roadside habitats.

Plant relative importance (*Pi*) size value was calculated for each species and *Pi* was calculated using the following formula: *Pi* = (relative cover + relative height + relative abundance)/3. The richness index, the Chao 1 richness index, the Shannon-Wiener diversity index, and the Simpson diversity index were selected to assess species diversity. The total number of species occurring within the sample was used as a measure of species richness (Wei et al., 2022).


$$Margalef:R_{m}=\left(s-1\right)/\ln N$$

$$Chao1=S_{obs}+\frac{n_{1}\left(n_{1}-1\right)}{2\left(n_{2}+1\right)}$$

$$Simpson:D=1-\sum_{i=1}^{s}P_{I}^{2}$$

$$Shannon-Wiener:H=-\sum_{I=1}^{S}\left(P_{i}\ln P_{i}\right)$$

Partial least squares discriminant analysis was performed based on the species importance values using the mixOmics package, and the results of the analysis were visualized with the ggplot2 package.

Nineteen bio30s meteorological data were extracted in ArcMap using the latitude and longitude of the sampling points (**Supplementary Table S1**). Pearson’s correlation analysis was used to explore the correlations between plant abundance and meteorological factors at different elevation gradients.

#### 2.3.2 Plant functional trait data collection and the functional diversity calculation

The functional traits of the plants were categorized into growth type, height, physiological characteristics, pollination, and dispersal mode. Ten plant functional trait indicators were used (**Table 1**), most of which were obtained from field measurements and surveys, and some of which were obtained in a literature review.

**Table 1:** Types and ways of obtaining plant functional traits.

Functional characteristic	Functional Characteristics Type	Acquisition method
Seed dispersal mode	1. Gravity propagation; 2. Wind propagation; 3. Automatic propagation; 4. Animal propagation.	FO
Pollination	1. Wind-borne; 2. Insect-borne.	FO
Light Habit	1. Negative; 2. Semi-negative; 3. Positive.	FO
Life history	1. Annual; 2. Biennial; 3. Perennial.	FO
Bloom period	Month of flowering	AI, FO
Florescence	Length of flowering period	AI, FO
Leaf shape	1. Round; 2. Oblong; 3. Lanceolate; 4. Elongate	FO
Leaf quality	1. Herbaceous; 2. Fleshy; 3. Leathery	FO
Average height	Standardization of measured data	FO
Total cover	Standardization of measured data	FO

FO, field observation; AI, access to information from references.

Plant functional diversity was measured by three dimensions, including functional richness, functional discretization, and functional homogeneity. After assigning values to the data for each functional trait of the plant, the functional diversity indices were calculated using the FD software package, including the functional richness index (*FRic*), the two-dimensional functional discretization indices (*FDiv* and *FDis*), and the functional homogeneity index (*Feve*).

#### 2.3.3 Vegetation community network structure

All species occurring in each treatment were included to construct the nodes of the network graph under that treatment. Spearman’s correlation analysis was calculated between two species with the species importance value and was used to construct the edges of the network graph for that treatment (Deng et al., 2012). Edges with a significance level greater than 0.05 and an absolute value of the correlation coefficient of less than 0.5 were excluded to clarify the constructed network graph, while the edges were constructed in the psych package. Based on the nodes and edges, the undirected randomized network graphs for each treatment were plotted using Gephi (v. 0.10.1). In the random network, the size of the average degree of connectivity was used to measure the strength of the species interactions in the community, and the higher the average degree of connectivity, the higher the complexity and stability of the vegetation community (Watts and Strogatz, 1998). Average clustering coefficient size was used to measure the level of community organization, and the larger the average clustering coefficient, the higher the level of community organization (Latapy, 2008).

#### 2.3.4 The relative contribution of elevation and distance to species diversity

As the plots were nested for each site and the sites were nested for the road, we used a linear mixed-effects model (LMM) to analyze the changes in the relative importance of the effects of elevation and proximity on the species α-diversity index, and the proportions of elevation, proximity, and their interactions were explained, where the fixed effects were elevation, distance, and site as a random effect. We used the R package lem4 (Bruelheide et al., 2021) to fit the LMM (**Table 2**). A hierarchical split was used for the proportion of explanations in each section (Lai et al., 2022). All analyses were conducted in R (v. 4.3.2; The R Foundation for Statistical Computing, Vienna, Austria).

**Table 2:** Results from LMM analyses with elevation and distance as fixed effects, and sites as the random effect.

Variables	Fixed effects	Sum Sq	DenDF	F value	*P* value	Deviation explained(%)
Richness	elevation	35.15	12.20	6.30	0.027	22.33
	distance	264.79	184.20	47.46	< 0.001	36.32
	elevation × distance	43.65	185.96	7.82	0.006	41.35
Shannon	elevation	1.51	12.16	8.49	0.013	45.67
	distance	3.99	184.52	22.41	< 0.001	25.67
	elevation × distance	0.23	186.53	1.31	0.254	28.66
Simpson	elevation	0.19	12.35	5.98	0.030	74.78
	distance	0.11	184.71	3.24	0.074	12.03
	elevation × distance	0.00	186.73	0.04	0.835	13.19
Chao 1	elevation	618.54	12.45	10.76	0.006	29.59
	distance	1624.66	185.30	28.26	< 0.001	32.67
	elevation × distance	619.77	187.66	10.78	0.001	37.74
cover	elevation	265.20	12.13	0.58	0.462	14.56
	distance	8712.90	183.88	18.99	< 0.001	41.41
	elevation × distance	1257.50	185.42	2.74	0.099	44.03
height	elevation	7.32	11.77	4.07	0.067	75.92
	distance	9.99	183.01	5.54	0.020	12.51
	elevation × distance	0.47	184.14	0.26	0.609	11.57

The last column reports the marginal R^2^ with 95% credible intervals of the model (row: effects) as well as the variance explained by elevation and distance.

## 3 Results

### 3.1 Effects of the road on grassland community diversity

**Figure 2:**
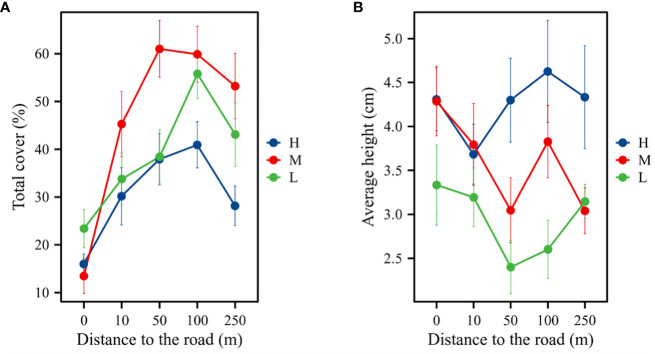
Changes in plant community height and cover along the distance gradient.

Meadows at five different distances from the road exhibited different plant community characteristics. The elevation, distance, and interaction between the two grasslands significantly affected plant community height and cover (*P*_A_ = 0.462, *P*_D_< 0.001, *P*_A × D_ = 0.099). At different distances from the road, vegetation cover was significantly lower at the slope (0 m) than at other distances (*P*< 0.05). The plant community cover of the three altitudinal gradients was unimodal with a significant increase followed by a significant decrease with increasing distance from the road (**Figure 2A**). Inconsistent trends were observed in plant community height at different elevations and distances (*P*_A_ = 0.067, *P*_D_ = 0.020). Plant community height decreased from 0 to 10 m and increased from 50 m to 100 m. The plant community heights at 50, 100, and 250 m were higher at high elevations than at the middle and low elevations (**Figure 2B**).

**Figure 3:**
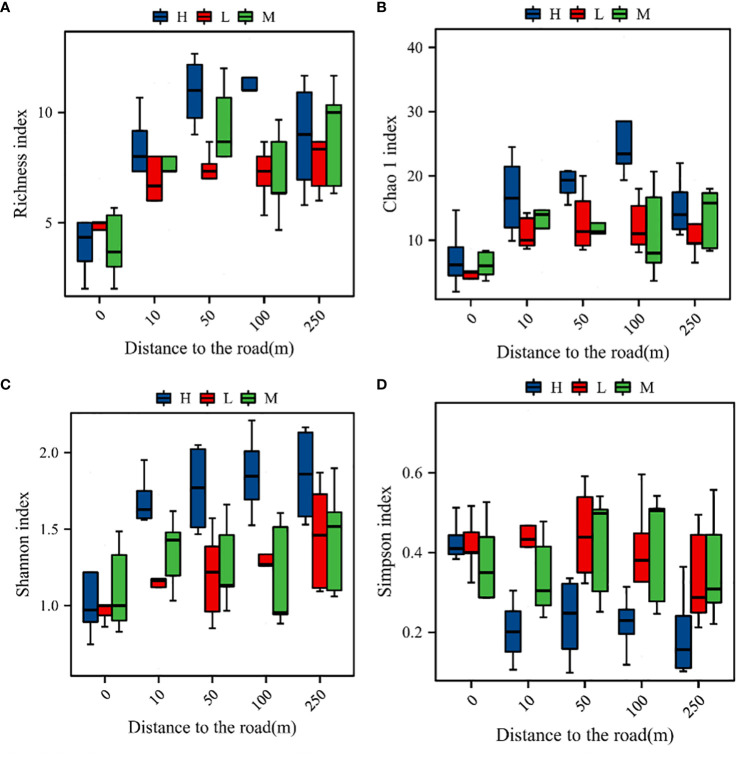
Species diversity indices of the plant communities. **(A)** Variation in the richness index with distance at different altitudinal gradients; **(B)** variation in the Chao 1 index with distance at different altitudinal gradients; **(C)** variation in Shannon diversity with distance at different altitudinal gradients; **(D)** variation in Simpson’s diversity with distance at different altitudinal gradients; H, M, and L represent high, middle, and low elevations, respectively.

Road distance and the altitudinal gradient had highly significant effects on the plant community richness index and the diversity index (*P*_A_ = 0.027, *P*_D_< 0.001, *P*_A × D_ = 0.006). The plant community richness index, the Chao 1 index, and the Shannon index were significantly (*P*< 0.05) higher at high elevations than at medium or low elevations. However, the difference in the species diversity indices between the mid- and low elevations was not significant (*P* > 0.05). The richness index, the Chao 1 index, and Shannon’s diversity index increased and then decreased with the increase of distance from the road (*P*< 0.05), being highest at a distance of 100 m. Simpson’s index had the opposite trend to Shannon’s index, being lowest at 50 m (**Figure 3**).

**Figure 4:**
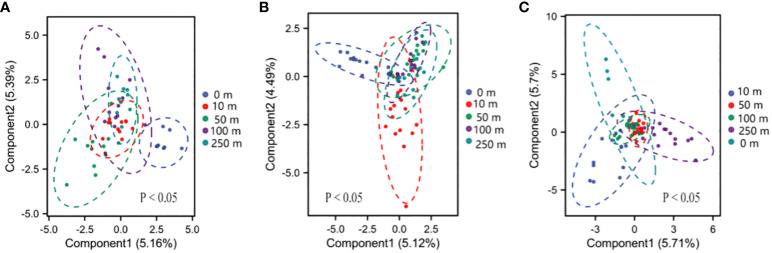
Beta diversity of the plant communities. **(A)** High-elevation gradient-biased least squares discriminant analysis (PLS-DA); **(B)** middle-elevation gradient-biased PLS-DA; **(C)** low-elevation gradient-biased PLS-DA.

The results of community Bray–Curtis heterogeneity analyses revealed significant differences in plant community composition across altitudinal gradients and at different distances from the highway. The 0-m distance had the highest Bray–Curtis heterogeneity at the high, medium, and low elevations. The difference in heterogeneity between 0 and 50 m was significant (*P*< 0.05) at high-elevations. The difference in heterogeneity at 0 and 10 m was significant (*P*< 0.05) at mid-elevation. The differences in heterogeneity were significant at 0, 10, and 250 m (*P*< 0.05, **Figure 4**) at low elevations.

**Figure 5:**
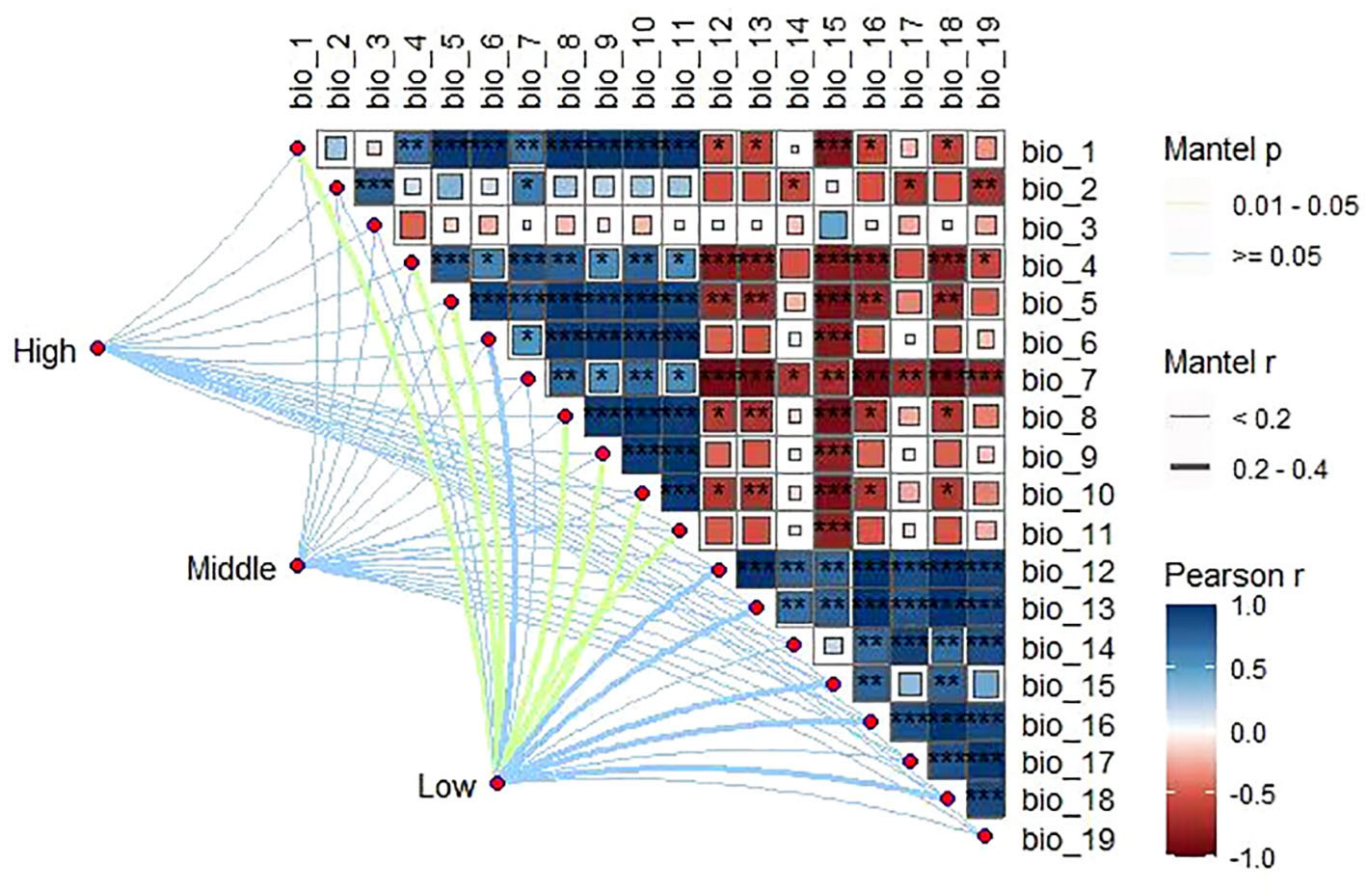
Correlation between species diversity and climatic factors at different altitudinal gradients (*, **, and *** indicate p ≤ 0.05, ≤0.01, and ≤0.001, respectively).

Among the meteorological factors extracted in this study, plant community composition across the altitudinal gradient was significantly and positively correlated (*P*< 0.01) with most of the temperature factors (BIO_1_–BIO_11_) and significantly and negatively correlated (*P*< 0.01) with the precipitation factors (BIO_12_–BIO_19_). In addition, the effects of different altitudinal gradients on climatic factors were consistent, with mean annual temperature and annual precipitation as the most important factors (**Figure 5**).

### 3.2 Effects of the road on plant functional diversity

**Figure 6:**
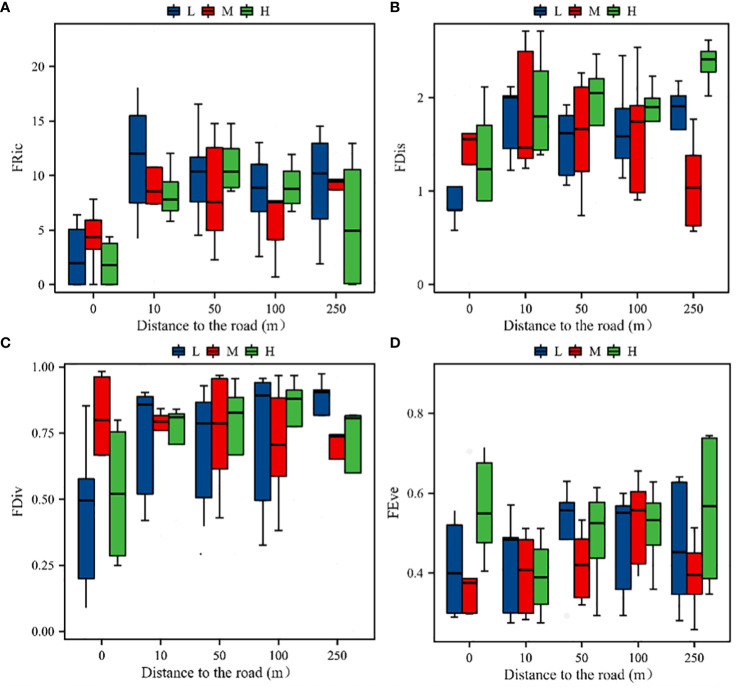
Species functional diversity indices of the plant communities. **(A)** Variation in the FRic index with distance at different altitudinal gradients; **(B)** variation in the *FDis* index with distance at different altitudinal gradients; **(C)** variation in the *FDiv* index with distance at different altitudinal gradients; **(D)** variation in the *FEve* index with distance at different altitudinal gradients; H, M, and L stand for high, middle, and low elevations, respectively.

Road distance had a highly significant effect on the plant community functional diversity indices (*P*< 0.01). However, the effect of the altitudinal gradient was not significant (*P* > 0.05), and the trends in the four functional diversity indices were consistent across the altitudinal gradient. Functional diversity richness (*FRic*) increased significantly (*P*< 0.05) with the increase of distance from the road at 10 m and then decreased slowly, which was consistent with the trend in the species richness indices. Functional diversity dispersion (*FDis* and *FDiv*) showed a single-peak trend of increasing and then decreasing, with lower levels at 0 and 250 m. None of the road distances had a significant effect on the evenness of functional diversity (*FEve*). However, the plant community functional evenness index was significantly (*P*< 0.05) higher at 0 m in the high-elevation zone than at other distances (*P* > 0.05). The degree of uniformity of the plant distribution was essentially the same at different distances (**Figure 6**).

### 3.3 Effects of the road on plant community network complexity

**Figure 7:**
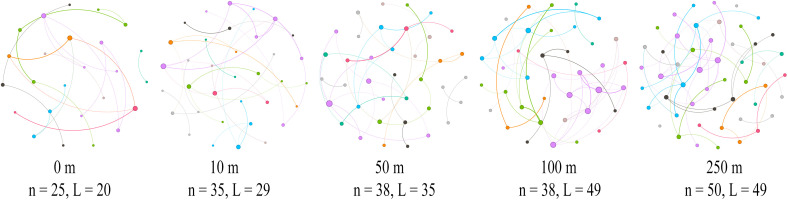
Plant community networks at different distances along the highway. Visualization of the constructed network at five different distances, with different modules shown in different colors.

**Figure 8:**
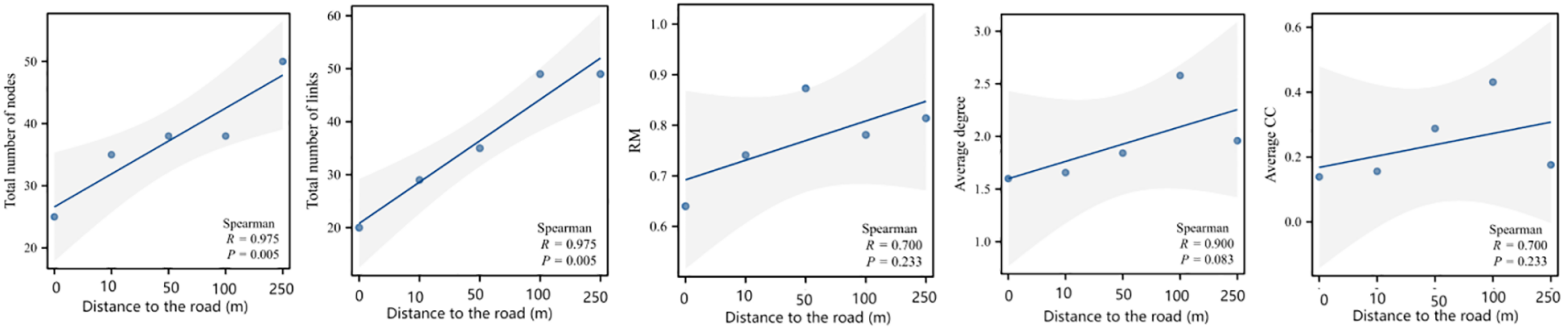
Changes in network topology with increasing distance, including the number of nodes, the number of connections, relative modularity, average degree, and the average clustering coefficient.

A co-occurrence network of the plant communities was constructed by comparing the mechanisms by which different distance gradients from the road affect the complexity of the co-occurrence network of the plant communities. This was accomplished following the importance values of the species at each distance, and various network topology parameters on distance were regressed (**Figure 7**). The total number of nodes (*R* = 0.975, *P* = 0.005), the total number of connections (*R* = 0.975, *P* = 0.005), and RM (*R* = 0.700, *P* = 0.005) were significantly higher as the distance from the road increased. The mean degree (*R* = 0.900, *P* = 0.083), mean clustering coefficient (*R* = 0.700, *P* = 0.233), and RM (*R* = 0.700, *P* = 0.233) all tended to increase (**Figure 8**).

## 4 Discussion

The importance of roads in promoting mountain biodiversity has been documented in a global database of research surveys showing the impact of mountain biodiversity on roads, which has been increasing since 2007 (Haider et al., 2022). However, grasslands at different distances from the road are affected differently. We considered the effects of roads on species richness and abundance, as well as the effects of roads on species functionality and stability. The grasslands at the side slopes were the most affected, reaching the farthest limit of impact at 100 m, while it was virtually unaffected at 250 m, and remained in its original grassland condition, with some sensitive species adapted to this growing condition with relatively low community stability.

### 4.1 Effects of the road on species diversity

During the construction and operation of a motorway, there will be varying degrees of impact on the soil environment and vegetation of the grasslands along the highway. As we predicted, grassland vegetation in areas close to the motorway was degraded to varying degrees. This result indicates that the closer the motorway is to a site, the lower the cover and diversity of the grassland vegetation, which is consistent with the results of many studies (Dörfer et al., 2013). This damage includes direct cutting and rolling of grass vegetation by construction and access vehicles (Wang et al., 2017). It may also be caused by soil erosion, which would change the topography on both sides of the motorway (Buckeridge and Jefferies, 2007). The impact of roads on roadside vegetation tends to be more severe due to the harsh environmental conditions in mountainous areas (Barni et al., 2007). The 0–10 m area of the road edge is highly affected by vehicles and road management, and the frequency of road construction and rehabilitation may compact the soil and result in water loss, which may affect plant growth and distribution and hence species richness and cover (Nepal and Way, 2007). These effects have resulted in the decline and disappearance of several sensitive species and have reduced biodiversity. Thus, species richness was lowest in the 0–10 m range, and the increase in species diversity at 10 m may have been due to the dust and water runoff particles from road management that extended into the soil and vegetation of many neighboring habitats, creating favorable conditions for plant growth (Müllerová et al., 2011).

Species richness was greatest at 50 m, and only slight variations in the number of species were observed at 50–100 m, until the threshold at 100 m, beyond which the number of species decreased. One possible explanation is that the 50 m distance represented the transition zone between the grassland edge and the core area, which is subject to road-induced disturbances and a core area relatively distant from the road. The increase in the diversity of the microenvironments for plant growth could increase plant species richness. Core communities, which are represented at distances of 100–200 m, were characterized by a sharp decline in the number of species probably because the new environments in these areas were suitable for a few specific species, and therefore there was lower species diversity.

Several global studies have shown that the richness of native species in the interior and roadside, as well as the assemblage of all species, forms a single-peak pattern with a maximum at lower mid-elevations (Haider et al., 2022). In contrast, our study showed that plant community diversity was significantly higher at high elevations than at low and middle elevations, and there was no significant difference in the diversity changes at low and middle elevations. However, this pattern changes along roads and for non-native species (Liu et al., 2021). Biodiversity patterns are largely shaped by natural factors, such as climatic gradients, and there is growing evidence that mountain biodiversity is affected by climate change (Lenoir and Svenning, 2024). Argentina (interior) and Australia (roadside) have negative correlations between elevation and native species richness (Rahbek, 2005). In contrast, Norwegian native species richness increases with elevation, possibly as a result of reduced shrub competition at higher elevations (Lembrechts et al., 2014). In this study, the difference in the elevation gradient was primarily due to the significant climatic differences along the Qinghai-Tibet Highway, with high temperatures in the south and low temperatures in the north based on latitude. It is windy along the Tibetan Plateau near latitude 32°N, with high winds throughout the year and decreased precipitation from southeast to northwest (Valdés et al., 2015). This climatic factor causes the plant communities along the Qinghai-Tibet Highway to become more diverse as the terrain rises.

### 4.2 Effects of the road on plant functional diversity and community stability

Many studies have shown that species diversity has a positive effect on community stability (Isbell et al., 2009). As communities become richer in species and more complex in structure, their stability increases (Campbell et al., 2011). Plant functional traits help to identify adaptive responses and resource allocation strategies in plants (Tilman, 1999). Exploring functional characteristics and functional diversity helps shed light on system stability, community structural mechanisms, and community productivity (Guittar et al., 2016). The results of this study indicate that plant functional diversity richness was significantly higher at 0 m than at the long-range, showing the same trend as species diversity, reaching a maximum at 100 m and decreasing at 250 m.

Analysis of the plant community network structure also showed that the grassland community was most unstable at 0 m. Road development projects require establishing surface processes at the project site, including logging, felling, and embankment construction (Rentch et al., 2005). Such processes disturb the topsoil and remove existing plant communities, turning the site into a barren ecosystem, and leading to long-term soil disturbance with consequences for community stability (Lamsal et al., 2018). As the distance from the road increases, the higher the network topology parameter value, the more stable the grassland community becomes, reaching a maximum at 100 m and then decreasing at 250 m. However, the grassland community was always more stable than the side slopes (0 m). Community stability increases with species diversity with road disturbance of a plant community, and plant community stability is maintained by plant functional diversity. The 100 m distance is critical in disturbed grassland communities, as this is the stage where habitats are most complex, species diversity is highest, and functional diversity reaches a saturation point. The road disturbance is virtually non-existent beyond 100 m; grassland habitats become native habitats, and community stability decreases. This study found that areas close to roads were likely to be colonized by exogenous species (Willard et al., 2007). In addition, plants on road edges change the community by changing basic drivers, such as temperature, moisture, light availability, and wind speed (Zurita et al., 2012). Consequently, the structure of the plant communities in the 0–10 m range is more prone to change, and community stability, distribution, species richness, and cover decrease.

### 4.3 Patterns of response of different species to road impacts

*Heliophilous* spp. and open habitat species likely colonize roads and side slopes (Ziegler et al., 2000), as the results of this study show that *Neotorularia torulosa* and *Plantago depressa* are endemic plants that grow on side slopes (0 m). *N. torulosa* prefers to grow in disturbed land, wasteland, sandy land, foothills, or stony clay slopes. *P. depressa Willd* is hardy, drought-tolerant, and adaptable, and has low soil requirements. Areas at the edge of roads are usually characterized by high levels of disturbance, the accumulation of gravel as vehicles pass, poor soil conditions and nutrients, low moisture conditions, and high light. Shade plants and scrub prefer the interior of a meadow. They are less tolerant of light and disturbances and are likely to grow inside undisturbed meadows. Sensitive and slow-growing species are vulnerable to roads, and they can be crowded out by more competitive species, retreating further away because of road and traffic construction. In this study, the endemic species at 250 m, such as *Lysimachia maritima*, grew in the lowlands of the plains, around reservoirs and lakeshores, as well as in the moist areas of mountain valleys (Liu et al., 2023). *Stellaria media* prefers a mild and humid environment (Oladeji and Oyebamiji, 2020). The greater the distance from the road, the less disturbed the grassland community will be, and the more these sensitive species, which require strict growing conditions, will survive (**Supplementary Figure S1**).

### 4.4 Potential factors affecting the road

Road distances that shape plant community diversity are strong potential drivers of climate (mean annual temperature, mean annual rainfall, and solar radiation). The destruction of vegetation also harms the road itself. Melting of permafrost under and near the road leads to subsidence of the road base. In addition, desertification of exposed soil occurs rapidly under the influence of strong winds and frequent freezing/thawing cycles. Therefore, the best way to protect vegetation is to apply mitigation measures at the planning and construction stages to keep initial damage to a minimum. Based on our results, we recommend that impacts on biodiversity be minimized by avoiding heavy vehicles that require larger roads or, when new roads are necessary, by methods that require specific planning in conjunction with long-term ecological monitoring and a proper impact modeling phase. Indeed, the geographic literature suggests that mitigation through appropriate road placement is critical to restoring and protecting alpine meadow core habitats, which, in turn, restores all ecosystem services needed for the human economy (Tattoni et al., 2011). Biodiversity can be further improved by the following steps: (1) accurate consideration of the road type and characteristics (Pereira et al., 2002) and historical dynamics may play a key role in detecting changes in biodiversity; (2) indicator taxa, such as animals, typical plants, insects, or birds, can also be used (Lawton et al., 1998). The importance of preserving core remnants of plant communities in the Tibetan Plateau landscapes requires a qualitative and quantitative understanding of the impact of human activities on the entire spectrum of species diversity. Future research on the effects of roads should focus on revealing the relative contributions of the different mechanisms that mediate the effects of roads, including fragmentation, vehicle-induced pollution, and changes in sedimentation rates (Petrin et al., 2023).

## 5 Conclusions

The impact of roads on species diversity is multifaceted. Increasing habitat diversity and species richness as well as the potential introduction of exotic species can affect plant functional traits, which, in turn, can negatively impact native species. This study investigated the effects of a road on roadside grassland plants at different distances. The results indicated that a road can severely degrade roadside grassland plant communities with reduced species diversity and functional diversity, resulting in low community stability and vulnerability to colonization by invasive alien species. These results will guide the restoration of grassland ecosystems along highways and the selection of road construction options in the Tibetan Plateau region.

## Data availability statement

The original contributions presented in the study are included in the article/**Supplementary Material**. Further inquiries can be directed to the corresponding authors.

## Ethics statement

Written informed consent was obtained from the individual(s) for the publication of any identifiable images or data included in this article.

## Author contributions

ZT: Writing – review & editing, Writing – original draft, Visualization, Validation, Software, Methodology, Investigation, Formal analysis, Data curation. XC: Writing – review & editing, Data curation, Visualization, Investigation, Funding acquisition. YW: Writing – review & editing, Investigation, Data curation, Funding acquisition. SW: Writing – review & editing, Formal analysis, Data curation. RW: Writing – review & editing, Formal analysis, Data curation. BY: Writing – review & editing, Formal analysis, Data curation. YY: Writing – review & editing, Formal analysis, Data curation. YK: Writing – review & editing, Funding acquisition, Formal analysis, Data curation. JQ: Writing – review & editing, Visualization, Software, Resources, Methodology, Funding acquisition, Formal analysis, Data curation.
