# Supplementary material for: The impact of the Qinghai-Tibet highway on plant community and diversity
Source: Front Plant Sci. 2024 Jun 28;15:1392924. doi: 10.3389/fpls.2024.1392924 (PMC11240119; doi:10.3389/fpls.2024.1392924)
Supplement: Supplementary file 1 [file DataSheet_1.docx]

Supplementary Material

##
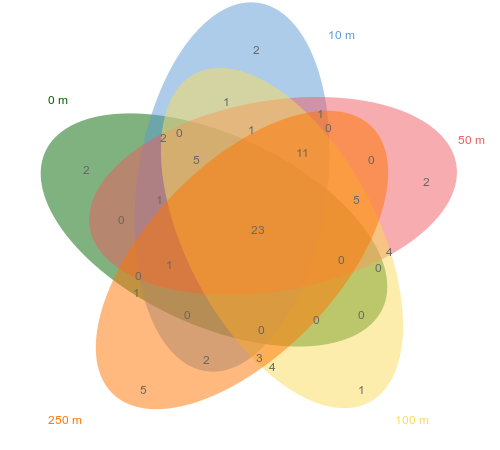
Supplementary Figures

## Supplementary Figure 1 Petal Venn diagram of plant compositions at different distances from the highway.
