# Supplementary material for: The impact of the Qinghai-Tibet highway on plant community and diversity
Source: Front Plant Sci. 2024 Jun 28;15:1392924. doi: 10.3389/fpls.2024.1392924 (PMC11240119; doi:10.3389/fpls.2024.1392924)
Supplement: Supplementary file 2 [file Table_1.docx]

Supplementary Material

## Supplementary Tables

**Table S1** 19 meteorological data from survey sites on the G109 national road

| site | 1 | 2 | 3 | 4 | 5 | 6 | 7 | 8 | 9 | 10 | 11 | 12 | 13 | 14 |
| --- | --- | --- | --- | --- | --- | --- | --- | --- | --- | --- | --- | --- | --- | --- |
| Lat | 36.4 | 31.6 | 35.3 | 35.5 | 35.0 | 34.3 | 32.2 | 33.7 | 34.0 | 31.9 | 34.6 | 33.3 | 33.0 | 32.6 |
| lon | 94.9 | 91.8 | 93.3 | 93.8 | 93.0 | 92.6 | 91.7 | 92.1 | 92.3 | 91.7 | 92.9 | 91.9 | 92.0 | 91.8 |
| bio1 | 5.5 | -0.8 | -4.5 | -4.4 | -4.4 | -3.7 | -2.3 | -3.8 | -4.2 | -2.5 | -5.3 | -4.3 | -6.8 | -5.8 |
| bio2 | 14.5 | 14.9 | 13.9 | 14.6 | 14.7 | 15.7 | 13.6 | 14.5 | 15.2 | 13.7 | 14.6 | 13.4 | 12.5 | 12.6 |
| bio3 | 35.2 | 40.7 | 37.9 | 38.2 | 39.6 | 40.5 | 37.8 | 38.9 | 40.0 | 38.6 | 39.6 | 37.2 | 35.2 | 35.7 |
| bio4 | 996.6 | 815.3 | 853.8 | 880.3 | 853.5 | 875.7 | 856.6 | 864.5 | 866.9 | 821.0 | 839.6 | 848.6 | 858.1 | 847.2 |
| bio5 | 25.0 | 15.4 | 12.6 | 13.3 | 12.7 | 14.1 | 13.8 | 13.4 | 13.1 | 13.4 | 11.7 | 12.3 | 9.8 | 10.3 |
| bio6 | -16.2 | -21.1 | -24.1 | -25.0 | -24.4 | -24.5 | -22.1 | -24.0 | -24.8 | -22.2 | -25.2 | -23.7 | -25.8 | -25.0 |
| bio7 | 41.2 | 36.5 | 36.7 | 38.3 | 37.1 | 38.6 | 35.9 | 37.4 | 37.9 | 35.6 | 36.9 | 36.0 | 35.6 | 35.3 |
| bio8 | 17.3 | 8.8 | 5.7 | 6.1 | 5.7 | 6.7 | 7.8 | 6.5 | 6.1 | 7.2 | 4.7 | 5.8 | 3.6 | 4.4 |
| bio9 | -4.2 | -9.1 | -14.1 | -14.4 | -14.1 | -13.7 | -11.1 | -13.5 | -14.0 | -10.9 | -14.7 | -13.8 | -16.2 | -15.2 |
| bio10 | 17.3 | 8.8 | 5.7 | 6.1 | 5.7 | 6.7 | 7.8 | 6.5 | 6.1 | 7.2 | 4.7 | 5.8 | 3.6 | 4.4 |
| bio11 | -7.2 | -11.1 | -15.2 | -15.5 | -15.1 | -14.6 | -13.1 | -14.5 | -15.0 | -12.8 | -15.7 | -14.8 | -17.3 | -16.3 |
| bio12 | 44.0 | 420.0 | 243.0 | 233.0 | 265.0 | 284.0 | 421.0 | 323.0 | 304.0 | 430.0 | 303.0 | 369.0 | 433.0 | 447.0 |
| bio13 | 12.0 | 106.0 | 63.0 | 59.0 | 70.0 | 80.0 | 111.0 | 91.0 | 86.0 | 111.0 | 82.0 | 101.0 | 119.0 | 120.0 |
| bio14 | 1.0 | 3.0 | 1.0 | 1.0 | 1.0 | 1.0 | 2.0 | 1.0 | 1.0 | 3.0 | 1.0 | 2.0 | 2.0 | 3.0 |
| bio15 | 83.4 | 112.4 | 111.1 | 109.3 | 113.5 | 117.3 | 115.2 | 117.9 | 117.9 | 113.7 | 114.6 | 115.4 | 116.2 | 114.5 |
| bio16 | 29.0 | 280.0 | 163.0 | 155.0 | 180.0 | 199.0 | 287.0 | 226.0 | 213.0 | 290.0 | 207.0 | 254.0 | 296.0 | 303.0 |
| bio17 | 3.0 | 9.0 | 3.0 | 3.0 | 3.0 | 4.0 | 8.0 | 5.0 | 5.0 | 9.0 | 4.0 | 7.0 | 9.0 | 10.0 |
| bio18 | 29.0 | 280.0 | 163.0 | 155.0 | 180.0 | 199.0 | 287.0 | 226.0 | 213.0 | 290.0 | 207.0 | 254.0 | 296.0 | 303.0 |
| bio19 | 3.0 | 9.0 | 4.0 | 4.0 | 4.0 | 5.0 | 9.0 | 6.0 | 6.0 | 9.0 | 5.0 | 8.0 | 10.0 | 11.0 |

Note: 1-14 refers to 14 points along the Qinghai-Tibet Highway
